# The little-known history of cleanliness and the forgotten pioneers of handwashing

**DOI:** 10.3389/fpubh.2022.979464

**Published:** 2022-10-20

**Authors:** Peter Poczai, László Z. Karvalics

**Affiliations:** ^1^Finnish Museum of Natural History, University of Helsinki, Helsinki, Finland; ^2^Museomics Research Group, Helsinki Institute of Life Science, University of Helsinki, Helsinki, Finland; ^3^Institute of Advanced Studies Kőszeg, Kőszeg, Hungary; ^4^Department of Cultural Heritage and Human Information Sciences, University of Szeged, Szeged, Hungary

**Keywords:** bacteria, handwashing, hygiene, infections, puerperal fever

## Abstract

Handwashing is a simple method for preventing the spread of pathogens. It is now common practice, but this was not always the case. Advocating for it often costed a doctor his career in the 1840s. Hospitals in the early 1800s had little idea of the significance of hygiene; thus, they were often mocked as disease-producing incubators or as “houses of death.” Many of the ill and dying were kept on wards with no ventilation or access to clean water; hospitals were found to offer only the most basic care. The mortality rate for patients admitted to hospital was three to five times greater than that for individuals cared for at home. Doctors did not routinely wash their hands until the mid-1800s, and they would proceed straight from dissecting a corpse to delivering a baby, providing the basis for the spread of puerperal fever. Despite advances in modern medicine, healthcare providers still face the issue of infection outbreaks caused by patient care. While the body of scientific data supporting hand hygiene as the key strategy to prevent the spread of pathogens is substantial, we highlight that achieving this crucial, long-awaited breakthrough was a hard task through history.

## Introduction

COVID-19, caused by the SARS-CoV-2 virus, has spread across the globe with no areas remaining unaffected by the pandemic (1, 2). The recommended strategy for keeping pathogens at bay is to wash our hands. Many nations have introduced measures preventing the spread of the virus and a manual on handwashing is provided by Ministries of Health in all countries (3–5). Thus, handwashing has received a great deal of attention as a basic preventative action carried out by the majority, since our transient skin flora (6) have an important role in the transmission of pathogens (6–8). The human skin is home to a wide variety of bacteria, viruses, fungi, and archaea, all of which play a significant role in the body's physiology through the skin microbiome (9). On average, a person's skin is home to around one thousand different kinds of bacteria, which together account for one billion bacteria per square centimeter (10). This equates to more than 1.6 trillion microbes spread throughout an average person's 1.8 square meters of surface area. The dynamics of hand microbial communities and the variables that have an effect on them are of major interest since hands are vital for the intrapersonal, interpersonal, and environmental transmission of microorganisms. It is possible for microorganisms with high pathogenic potential but short-term survival rate on the skin to spread if we avoid thoroughly washing our hands; consequently, hygiene programs have been running in all healthcare systems urging 20 s of handwashing (11). In contrast, there was little awareness of the significance of hand hygiene throughout the first half of the 1800s. In 1825, visitors to St George's Hospital in London found mushrooms and maggots flourishing in the wet, filthy blankets of a patient (12). At the same time surgeons, rather than cleaning their hands after dissection, would go from cutting up a corpse to assisting with a birth. No wonder then those hospitals and maternity clinics were referred to “*[as] real houses of death*” rather than places of treatment and rehabilitation (13). Mortality rates in hospitals skyrocketed and the methodology accounting for deaths was as reliable as a “*hundred apples divided by fifteen red herrings*” (14). Medicine was in its infancy and linking pathogens to infections was a huge discovery to be made. In the 1800s, handwashing was not medical common sense; it was outrageous. In fact, advocating for it often cost a doctor his career at that time. But how did handwashing become a central topic in pathogen control? Herein, we explore the history of handwashing in medicine and review how this behavior has been recognized and accepted as part of antisepsis and hygiene.

## Handwashing: Myth, magic, and religion

**Figure 1:**
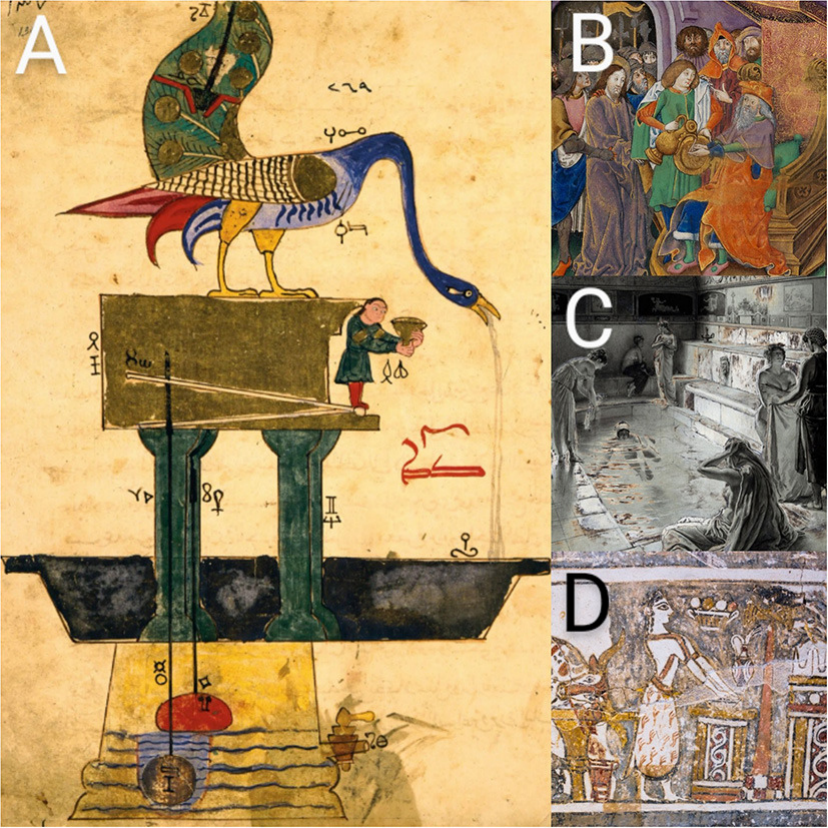
**(A)** *The Book of Knowledge of Ingenious Mechanical Devices* (1206) by the Mesopotamian polymath Ibn al-Razzāz al-Jazari (1136–1206), depicts a peacock basin automaton for ritual hand washing. We do not know with certainty that al-Jazari's device was ever actually constructed. Photo courtesy of the Cleveland Museum of Art. **(B)** Handwashing was also associated with the removal of sin and misfortune. The *Peniarth Manuscript* ca. 1503 depicts Christ before Pilate, with Pilate washing his hands (MS 481D). Photo courtesy of the National Library of Wales. **(C)** The Roman bath in the Strand, London, as used in the time of the Roman occupation of Britain. Colored pencil drawing by Fortunino Matania from 1922. **(D)** The unique representation on the Hagia Triada Sarcophagus (ca. 1400 BCE) depicts ritual handwashing after sacrificing a bull.

Physical cleansing was motivated by the so-called “Macbeth effect” for religious reasons (15) rather than to prevent infections from spreading. For centuries, religious and magical rituals included handwashing as an essential component (Figure 1). Asterius was struck dead by a thunderbolt in Greek mythology because he approached the altar of Zeus with unwashed hands (16). Hygieia (Greek υ,γíε*ια* = *hugieia*) was the goddess of health and cleanliness in Greek as well as Roman mythology (17). Until the hands were bathed in a live stream, Romans would not allow anybody to handle sacred things with tainted hands (18). According to Jewish belief, unwashed hands can let devils into the eyes, nose, and ears (19). Handwashing before religious rituals was common in both Islamic and Christian cultures, but it was also thought to cleanse the body of sin, misfortune, evil, and the burden of guilt (20, 21). The mystical and religious roots of handwashing may be a reflection of our society's obsession with cleanliness. Contemporary psychopathologies related with handwashing may be divided into two broad categories: those that are founded on anxiety and those that don't have a specific aim (22).

Prior to any genuine knowledge of cleanliness, and in addition to the religious and symbolic implications of handwashing, the practice of public bathing was acquired from Ancient Rome and Eastern Cultures and became a popular social pastime throughout the 14th century (23). During this period, bath houses were beautiful social gathering places, but they were eventually transformed into brothels (24). By the early 17th century, this form of the bath houses had vanished. According to McLaughlin (25), “*the decline of the baths was due to their association with promiscuity and prostitution*.” Handwashing was undertaken after the fall of public bath houses and before the invention of the fork, since the hands were used to eat from a shared dish in the center of the table (26). Handwashing cleanliness and table etiquette were said to be repulsive by our standards during these early periods. This was a time when sanitation was deplorable. Soap existed, although it was rarely used (26). Poor hygiene was not restricted to the lower classes; the extremely wealthy were regarded as “nasty and hideous” (27). Europe had acquired an eastern cultural habit, modified it, distorted it, and destroyed it (25). But how has handwashing been linked to hygiene and disinfection throughout the history of cleansing?

## The lady with the lamp and the twin stars of handwashing

Moses ben Maimon, also known as Maimonides (1138–1204), recognized the need for handwashing for excellent hygiene in medicine as early as 1199 (28). He began washing his hands after handling a sick person, dismounting a horse, and treating patients. Even though history would show him to have been correct, his method of disinfection was not well received by his peers (29). Worse, until the germ theory was accepted in the early 20th century, there was fierce hostility to this new explanation of illness and disinfection practice (30). Unlike ceremonial handwashing, which dates back thousands of years, handwashing for disinfection is a recent concept. Girolamo Fracastoro's (ca.1478–1553) “spore theory” (31) was competing with Galen's miasma (μíα*σμα*, Ancient Greek for *pollution*) concept (32). In 1546, Fracastoro postulated that infections are caused by small particles called “spores” that may be transferred from one person to another (33). These spores can spread disease by direct touch, indirect contact, or even without contact over large distances. Though, the term “spores” may refer to chemicals rather than biological organisms in Fracastoro's writings. Interestingly, he was also a renowned poet, and the term *syphilis* is taken from his 1530 poem *Syphilis sive morbus gallicus* (“*Syphilis or The French Disease*”), which is about a young shepherd named Syphilus who tended King Alcinous' herds.

**Figure 2:**
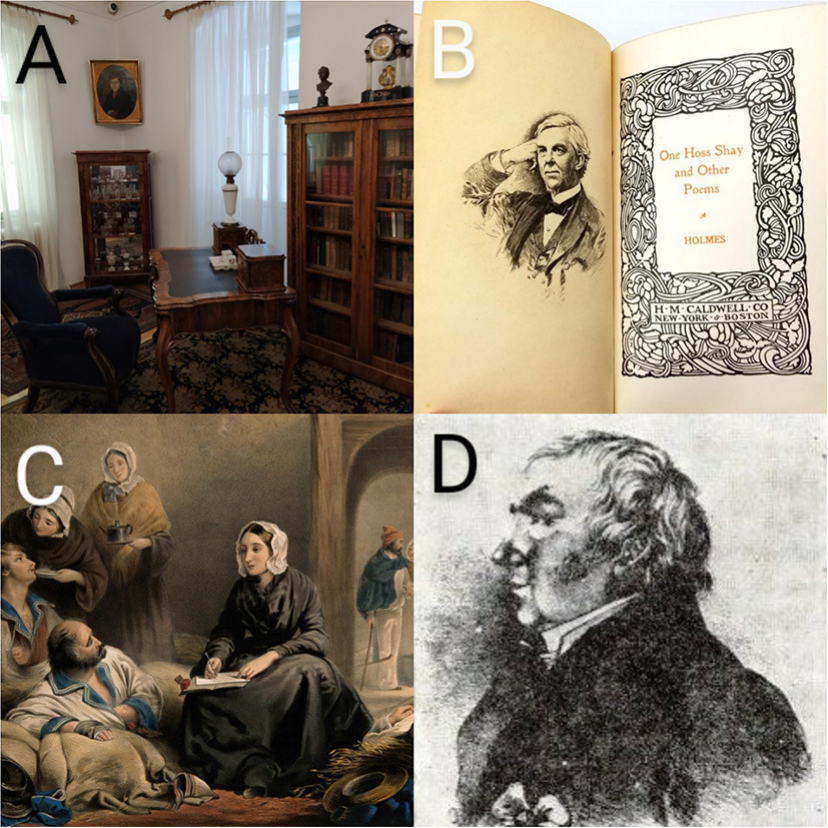
**(A)** The memorial room of the HNM Semmelweis Museum, Library and Archive of the History of Medicine, rearranged from the estate and furniture of Ignác Semmelweis. Photo by P. Poczai. **(B)** Holmes was a physician by profession but gained popularity as a writer; he was one of the nineteenth century's most esteemed American poets. **(C)** Florence Nightingale visiting patients on the colored lithograph of R. Burgess. Portrait courtesy of the Wellcome Institute, London (No. 2151.25). **(D)** The only surviving depiction of János Zsoldos.

By the end of the 19th century, academics believed that diseases—and even obesity—were caused by inhaling corrupted air (34). The application of antisepsis came under scrutiny in connection with puerperal fever (childbed fever, puerperal sepsis), coined by Edward Strother (1675–1737) in 1716 (35), as highly fatal epidemics swept Europe and the UK (36). The historical debate over who was the first to identify the contagious nature of puerperal fever continues (37), with most medical historians crediting two 19th century physicians: the American Oliver Wendell Holmes (1841–1935) (38–41) and the Hungarian Ignáz Fülöp Semmelweis (1818–1865) (32–45). In 1843 Holmes proclaimed insistently that “*doctors were agents of death”* unless they washed their hands and clothing to avoid transmission of puerperal fever (46). While Semmelweis was the first to statistically prove the contagiousness of the illness in 1847, he refused to publish his results (47); this was done by his students (48–51) until he finally published his book in 1861 (52). He demonstrated that the hospital wards open to medical students and physicians had a much higher mortality rate than those open only to midwives (53). His answer was to demand preventative handwashing in a chloride and lime solution first made by Labarraque (1777–1850) (54) to eliminate “*cadaverous particles*.” This was one of the first times that antisepsis was used but Semmelweis faced serious persecution. Although Holmes was not a practicing physician, his views were supported by many (36, 55), while his opponents such as Meigs (1792–1869) cynically branded his work as “*childish and sassy dreaming*” by turning his literary and poetic success against him (56, 57) (Figure 2).

Florence Nightingale (1820–1910) “*The Lady with the Lamp*” should also be credited with recognizing the need for excellent cleanliness. She was the driving force behind the mid-nineteenth century hospital reform movement. She rose to fame because of her contributions to the Crimean War (1853–1856). At that time, it was customary for two soldiers to die of sickness for every soldier slain on the battlefield, e.g., dysentery, diarrhea, typhoid, malaria. Soldiers from small, secluded rural communities who had never experienced childhood diseases such as measles and mumps exacerbated the issue (58). They lacked immunity to these prevalent and dangerous illnesses on the battlefield. The overcrowded and filthy circumstances in the hospital exacerbated outbreaks of these illnesses. Florence Nightingale's humanitarian endeavor at the Scutari Barracks Hospital during the war was a resounding success, and she was able to persuade the world of the importance of increasing hygiene and sanitation, as well as having professionally trained nurses care for patients in hospital wards. Nightingale (59) urged nurses to wash their hands and faces regularly throughout the day, demonstrating a long-standing appreciation for the efficacy of hand hygiene. By the time she returned to England, she was a national hero.

The common conception of Nightingale as a romantic heroine disregarded her educational accomplishments. Nonetheless, the Nightingale legend had a lasting educational influence. It popularized nursing training, resulting in the creation of a new career for women. Even though Nightingale's mythology has remained an integral element of nursing culture worldwide, it has impeded our ability to comprehend her in greater depth (59).

In the late 19th century, the germ theory, stating that certain diseases are caused by the invasion of the body by microorganisms, gradually started to gain acceptance thanks to the French chemist Louis Pasteur (1822–1895) (60). During his investigations in the 1860s, Pasteur demonstrated that food spoils due to unseen bacterial infection, not by spontaneous creation (61).

The 1861 publication of Pasteur's germ theory of diseases was a turning point in the development of medicine, but advances took many years to take effect. Joseph Lister (1827–1912) recognized the surgical implications of Pasteur's research on the function of microbes in fermentation; in 1865 he used a spray made of carbolic acid on wounds, dressings and surgical tools (62). In 1867, Lister suggested that his method of antisepsis was responsible for the remarkable decreases in surgical and overall hospital infectious disease mortality (63). In 1876, Robert Koch (1843–1910) expanded on Pasteur's findings and conclusively established that a particular germ could cause a specific disease (64). During the era often called the “microbe hunt” he correctly discovered the germs responsible for anthrax, septicemia, tuberculosis, and cholera. Lister's approach to surgery spread swiftly, thanks in part to his instruction of hundreds of medical students and also to his astute patronage. Together with germ theory they produced the desired effect, they diminished wound morbidity and patient mortality. These contributions were secured for future generations and a surgical revolution had begun.

## Before Holmes: Pre-Semmelweisian pioneers

The idea of handwashing and hygiene developed gradually (Table 1). The unsung pioneers had to demonstrate real courage in speaking the truth in the face of opposition and mounting prosecution. The fact that puerperal fever is infectious was not discovered only by Semmelweis and Holmes. Semmelweis disputed the infectiousness of puerperal fever in the sense that it may be a source of contagion that causes the same illness in another person (52, 53). He wrote “*[it] is not a species of disease; puerperal fever is a variety of pyemia*” (53). Such nature of puerperal fever did not go completely unnoticed among physicians following its Hippocratic description (36). In 1751, John Burton (1710–1771) could have been the first to propose that the illness might be infectious (65, 66). William Smellie (67) and Thomas Cooper (68) agreed with Burton but failed to explain its etiology. In 1772, John Leake (1729–1792) also believed in its contagious nature (69), and the following year, Charles White (1728–1813) urged mothers who had just given birth to keep their surroundings clean (70); he did not stress hygiene, this was hinted by Joseph Clarke (1758–1834), who suggested separating patients and cleaning wards in 1790 (71). Alexander Gordon (1752–1799), an ex-naval surgeon, described cases of puerperal sepsis in Aberdeen, Scotland in 1789, and advocated disinfection and handwashing for its prevention (72, 73). Thomas Denman (1733–1815) in 1801 regarded Gordon's theory as “*fully proved*” (74).

**Table 1:** Landmark contributions in hand hygiene and infection control from 1546 to 1867.

**“Puerperalia” (Gynecological context)**			**General (surgical) context**		
			1867	Joseph Lister (1827–1912)	*Antiseptic surgery*
1847	Ignáz Fülöp Semmelweis (1818–1865)	Mandatory chlorine *handwashing* in Vienna General Hospital			
			1853	Florence Nightingale (1820–1910)	Implemented hand washing and other hygiene practices in British army hospitals
1843	Oliver Wendell Holmes (1809–1894)	“*physicians with unwashed hands are responsible for transmitting puerperal fever from patient to patient”*			
			1820	Antoine Germain Labarraque (1777–1850)	Use of sodium-hypochlorid as disinfectant
1814	Zsoldos János (1767–1832)	“*physicians and midwives, wash your hands with lime and soapy water before and after childbirth”*			
			1809	Zsoldos János (1767–1832)	Handwashing rules, disinfection of medical equipment with camphoric burnt wine
1795	Alexander Gordon (1752–1799)	Described cases of puerperal sepsis, advocating for disinfection and handwashing for its prevention			
1751	John Burton (1710–1771)	Puerperal fever is a contagious disease			
			1546	Girolamo Fracastoro (1478–1553)	Infection could be passed on *via* hands and clothes

Another surgeon, János Zsoldos [*Z*oldo*S*] (1767–1832) from Veszprém County, Hungary, also stressed the importance of antisepsis. Working in the battlefields during the Napoleonic Wars in 1809 he applied handwashing and disinfected his medical equipment with “*camphoric burnt wine*” (75). He observed that this procedure “*prevented the transmission of sticky maladies*” (76). Thus, he made it clear for physicians and midwives in 1814 to wash their hands with lime and soapy water before and after childbirth or even treating a wound (77). Publishing his guidelines three decades earlier than Semmelweis and Holmes he could be considered as one of the forgotten forerunners of handwashing, fighting for necessary surgical hand rubbing in hospitals. His book *Rules of Procedures (Diaetetika)* (77) was later published in four editions (78, 79) including two poetic adaptations: one by his brother Jakab Zsoldos (80) and later by Gerzson Fodor (81). A passage from *Diaetetika* in the verse of Fodor reads as follows:

Doctors must wash themselves and their hands always,

and not touch a festering, infected wound, in any case—

especially with hands that are also with wounds.

And should often clean their barber's tools.

Midwives — do not reach for one giving birth

with wounded hands, but cleanse them first—

with vinegar and soap, rub them with lye, with ash,

and only visit your next patient after that.

Then rinse your hands well in bran water,

Start and finish your work clean, forever.

However, it is almost incomprehensible that the teachings of Zsoldos were not adopted by physicians, since the poems were very popular (79). The standards and actual practices of medical and paramedical staff of the time regarding handwashing cannot be reconstructed from written sources. However, indirect evidence can help to clarify whether the principles identified by Zsoldos were translated into the daily medical practice in the Women's Hospital (*Asszony Ispotályi Intézet*) what he established in Pápa in 1816 (79). The hospital's inventory records from 1816, the year of its foundation, could provide a partial answer (82). The hospital inventory makes a clear distinction between the metal utensils provided for patients and the textile linen used after cleaning them (linen “towelettes” or “washcloths”) and the handwashing bowls and hand towels which are part of the medical equipment (83). It is clear from the inventory that the hand towels and rinsing bowls (described as “cephalic pots” or “wash basins”) were not only used in the service rooms but also in the wards. It should also be mentioned that according to the records, discarded textiles were transferred to medical equipment as “wound cleaning cloths” or “wound wiping cloths”, while copper bowls, pots and tubs were regularly cleaned and replaced.

The nomenclature used in the inventory indirectly proves that Zsoldos not only considered it essential at a theoretical level to educate physicians and nurses to wash their hands in a thorough and responsible way, but also made handwashing a regular practice in the hospital he founded. According to hospital records during the first quarter of a century, there were a few deaths which caused a great stir, but relatively fewer deaths were recorded in Zsoldos's hospital. The pioneering practice of hygiene may have played some part in this.

## Conclusions

Handwashing has been recognized as important in medicine for almost seven centuries, but widespread adoption has been developing slowly (84). During the early history of medicine, this practice was not widely acknowledged, and it was first connected with religious and magical practices. It later became a communal activity, but only lately has handwashing been linked to antisepsis and cleanliness. Since the introduction and validation of the historical idea, great progress has been made in the application of recommendations for the improvement of hand hygiene. For instance, it has been demonstrated that effective hand hygiene reduced fatalities from respiratory and diarrheal infections in children under five by 21 and 30%, respectively (85). Yet, by 2021, an estimated 2.3 billion people worldwide will be unable to wash their hands at home with soap and water, and one-third of the world's health institutions would lack hand hygiene tools at the point of service (85, 86). Meanwhile, over half of the world's schools lack essential sanitary services, affecting 817 million students (85). Thus, the *Hand Hygiene for All* program and the *World Hand Hygiene Day* was established by UNICEF, WHO, and other partners in an effort to channel enthusiasm surrounding hand hygiene into long-term, sustainable change (85, 87).

Unfortunately, early forerunners of antisepsis were overlooked and they did not see the results of their work. Semmelweis, like Holmes, was left to make the same painstaking discovery on his own. However, Holmes quoted Gordon to support his claims (46) and while repeating his views in 1855 also referred to Semmelweis (88). We can only speculate as to whether Semmelweis knew about the work undertaken overseas. A ban on international literature did no good for scientific work in Central Europe between 1820 and 1848 (89–91). At least he might have read some of them around 1860 since he responded to criticism in great detail (92). Semmelweis might have been aware of the book by Zsoldos, which was part of the medical curriculum (79, 93). What we know for certain is that Semmelweis was eventually redeemed but only after he had been driven to insanity (94). Similar to Semmelweis, Gordon was also persecuted (73). Several factors combined could have led to the rejection of the hypothesis of the early pioneers that stressed a link between hygiene and infections, but the major reason was the primal human behavior dominant in the medical community of the time, which favored sticking to existing beliefs and rejecting new ideas that contradicted them (95). This tendency is now often referred to as the Semmelweis reflex (96), which is still dominant in the current pandemic in the refusal to wash our hands (97).

After describing the development of the handwashing principle in a more detailed way, it is also necessary to understand the nature of the roadblocks to early adaptation. It can be an important contribution in revealing efforts to identify historical patterns in the growth of medical knowledge and innovation. We already know that the efficacy of new knowledge depends on the feedback generated by its application in relation to specific problems, and social understanding is central for both the accumulation and the recombination of knowledge (98). However, we still do not have metrics to estimate the critical mass of receivers/followers, which is necessary for the reproduction or multiplication of the new medical knowledge. Further research needed to unfold the rules and quantifiable aspects of the social framework of medical information flow (99), the preconditions of accumulation and augmentation of medical knowledge.

Handwashing is a socially influenced behavioral phenomenon (100). It is comparable to other preventative habits in that there are no immediate positive or negative repercussions to participating or avoiding the activity. As a result, it lacks intrinsic reinforcing qualities. Despite its ancient historical origins in magic and religion, the behavior has yet to become regular in many circumstances (100). The forerunners of handwashing were met with nothing but ignorance and mockery. Let us try to improve our hand hygiene and think about them as we wash our hands.

## Data availability statement

The original contributions presented in the study are included in the article/supplementary material, further inquiries can be directed to the corresponding author.

## Author contributions

The study was conceived and all investigation related to the study was carried out by LK and PP. Handwritten documents were transcribed by LK. Further resources were provided to the study by PP and LK. Funding was acquired by PP, who carried out all project administration and wrote the original draft. All authors have reviewed, edited, and approved the final version of the manuscript.

## Funding

Open access publication was funded by the Finnish Museum of Natural History.

## Conflict of interest

The authors declare that the research was conducted in the absence of any commercial or financial relationships that could be construed as a potential conflict of interest.

## Publisher's note

All claims expressed in this article are solely those of the authors and do not necessarily represent those of their affiliated organizations, or those of the publisher, the editors and the reviewers. Any product that may be evaluated in this article, or claim that may be made by its manufacturer, is not guaranteed or endorsed by the publisher.

## References

[1] Colella JP Bates J Burneo SF Camacho MA Bonilla CC Constable I. Leveraging natural history biorepositoties as a global, decentralized, pathogen surveillance network. PLoS Path. (2021) 17:e1009583 10.1371/journal.ppat.1009583 34081744 PMC8174688

[2] Roberts DL Rossman JS Jarić I. Dating first cases of COVID-19. PLoS Path. (2021) 17:e1009620 10.1371/journal.ppat.1009620 34166465 PMC8224943

[3] Ministry of Health New Zealand. Good Hygiene Recommendations on Handwashing. Available online at: https://www.health.govt.nz/your-health/healthy-living/good-hygiene/hand-washing (accessed September 23, 2022).

[4] Centers for Disease Control Prevention (CDC). Handwashing in Communities: Clean Hands Save Lives. Available online at: https://www.cdc.gov/handwashing/index.html (accessed September 23, 2022).

[5] Ministry of Health Saudi Arabia. Hand Hygiene. Available online at: https://www.moh.gov.sa/en/HealthAwareness/EducationalContent/PublicHealth/Pages/Handwashing.aspx (accessed September 23, 2022).

[6] Rotter M. Hand washing and hand disinfection. In: Mayhall CG, editor. Hospital Epidemiology and Infection Control. 2nd ed. Philadelphia, PA: Lippincott Williams & Wilkins. (1999). p. 1339–1355.

[7] Pittet D Boyce JM. Hand hygine and patient care: pursuing the Semmelweis legacy. Lancet Inf Dis. (2001) 1:9–20. 10.1016/S1473-3099(09)70295-6

[8] Savolainen-Kopra C Haapakoski J Peltola PA Ziegler T Korpela T Anttila P. Hand washing with soap and water together with behavioural recommendations prevents infections in common work environment: an open cluster-randomized trial. Trials. (2012) 13:10 10.1186/1745-6215-13-10 22243622 PMC3296604

[9] Byrd AL Belkaid Y Segre JA. The human skin microbiome. Nat Rev Microbiol. (2018) 16:143–55. 10.1038/nrmicro.2017.157 29332945

[10] Knackstedt R Knackstedt T Gatherwright J. The role of topical probiotics on wound healing: a review of animal and human studies. Int Wound J. (2020) 17:1687–94. 10.1111/iwj.13451 32869480 PMC7949352

[11] CDC (Centers for Disease Control Prevention). Coronavirus Disease 2019 (COVID-19): FAQ on Hand Hygiene. Available online at: https://www.cdc.gov/coronavirus/2019-ncov/infection-control/hcp-hand-hygiene-faq.html (accessed March 26, 2020).

[12] Anonymous Hospital Reports. St. George's hospital. Lancet. (1825) 5:142–3. 10.1016/S0140-6736(02)91261-4

[13] Edwards KT. Childbed fever: a scientific biography of Ignaz Semmelweis. JAMA. (1994) 272:1871–2. 10.1001/jama.1994.0352023008104925996397

[14] Anonymous. Reviews. Notes on hospitals. Med. Tim. Gaz. (1826) 30:129–30.

[15] Zhong C-B Liljenquist K. Washing away your sins: threatened mortality and physical cleaning. Science. (2006) 313:1451–2. 10.1126/science.1130726 16960010

[16] Potter J. Archaeologia Graeca. 3rd ed. London: Thomas Tegg & Son (1837). p. 224.

[17] Norman NJ. Asklepios and Hygieia and the Cult Statue at Tegea. Ame J Archaeol. (1986) 90:429. 10.2307/506027

[18] Smith V. Clean: A History of Personal Hygiene and Purity. Oxford: Oxford University Press (2008). p. 102–16.

[19] Ashenburg K. The Dirt on Clean: An Unsensitized History. Toronto, ON: Vintage Canada (2008). p. 54–55.

[20] Abdel-Khalek AM. Religiosity and well-being in a Muslim context. In: Kim-Prieto C, editor. Religion and Spirituality Across Cultures. London: Springer (2014). p. 75–7.

[21] Al-Tawfiq JA Memish ZA. Religion and hand hygiene. In: Pittet D, Boyce JM, Allegtanzi B, editors. Hand Hygiene: A Handbook for Medical Professionals. Oxford: Wiley Blackwell (2017). p. 217.

[22] Matsunaga H Mukai K Yamanishi K. Acute impact of COVID-19 pandemic on phenomenological features in fully or practically remitted patients with obsessive-compulsive disorder. Psy Clin Neurosci. (2020) 74:565–6. 10.1111/pcn.13119 32697002 PMC7404884

[23] Shephard RJ. A History of Health and Fitness: Implications for Policy Today. Oxford: Springer (2018). p. 347.

[24] Agnew J. Healing Waters: A History of Victorian Spas. Jefferson, MO: McFarland & Company (2019).

[25] McLaughlin T. Coprophilia or Peck of Dirt. London: Cassel & Company (1971).

[26] Krebs RE. Groundbreaking Scientific Experiments, Inventions and Discoveries of the Middle Ages and the Renaissance. London: Greenwood Press (2004). p. 229–32.

[27] Ward P. The Clean boy: A Modern History. Québec: McGill-Queen's University Press (2019).

[28] Pearson A. Historical and changing epidemiology of healthcare-associate infections. J Hosp Inf. (2009) 73:296–304. 10.1016/j.jhin.2009.08.016 19925942

[29] Eisenberg RL. Essential Figures in Jewish Scholarship. Plymouth: Jason Aronson (2016).

[30] Farley J. Parasites and germ theory of disease. Hosp Pract. (2016) 27:175–96. 10.1080/21548331.1992.11705490 1522158

[31] Crawford DH. Deadly Companions: How Microbes Shaped Our History. Oxford: Oxford University Press (2007).

[32] Byrne JP. The Black Death. Westport, CT: Greenwood Press (2004). p. 44.

[33] Nutton V. The reception of Fracastoro's theory of contagion: the seed that fell among thorns? Osiris. (1990) 6:196–234. 10.1086/368701 11612689

[34] Halliday S. Death and miasma in Victorian London: an obstinate belief. Brit Med J. (2001) 323:1469–71. 10.1136/bmj.323.7327.1469 11751359 PMC1121911

[35] Porter IA. Alexander Gordon, MD of Aberdeen 1752–1799. Edinburgh: University of Aberdeen by Oliver and Boyd (1958).

[36] Peckham CH. A brief history of puerperal infection. Bul Inst Hist Med. (1935) 3:187–212.

[37] Lowis GW. Epidemiology of puerperal fever: the contributions of Alexander Gordon. Med Hist. (1993) 37:399–410. 10.1017/S0025727300058749 8246645 PMC1036777

[38] Cullingworth CJ. Oliver Wendell Holmes and the Contagiousness of Puerperal Fever. London: Henry J. Glaisher (1906) pp. 1–8. 10.1136/bmj.2.2340.1161 PMC2322679 20762355

[39] Litoff JB. American Midwives: 1860 to the Present. Westport Conn.: Greenwood Press (1978). p. 19.

[40] Leavitt J. Brought to Bed: Childbearing in America, 1750 to 1795. New York, NY: Oxford University Press (1986). p. 155.

[41] Wertz RW Wertz DC. Lying-in: A History of Childbirth in America. New Haven: Yale University Press (1986). p. 120–123.

[42] Sinclair WJ. Semmelweis, His Life and Doctrine, a Chapter in the History of Medicine. Manchester: University Press (1909).

[43] Carter KC. Semmelweis and his predecessors. Med Hist. (1981) 25:57–72 10.1017/S0025727300034104 7012475 PMC1138986

[44] Wynder EL. Ignaz Phillip Semmelweis. Prev Med. (1974) 3:574–80. 10.1016/0091-7435(74)90022-X 4612515

[45] Newsom SWB. The history of infection control: Semmelweis and handwashing. Brit J Inf Contr. (2001) 2:24–5 10.1177/175717740100200410

[46] Holmes OW. The contagiousness of puerperal fever. New Engl Quart J Med. (1843) 1:503–30.

[47] Reid RW. Microbes and Men. New York, NY: Saturday Review Press (1975). p. 37.

[48] Hebra F. Höchst wichtige Erfahrungen über die Aetiologie der an Gebäranstalten epidemischen Puerperalfieber. Zeits Gesells Ärzt Wien. (1847) 4:242–4.

[49] Hebra F. Fortsetzung der Erfahrungen über die Aetiologie der in Gebäranstalten epidemischen Puerperalfieber, *Zeits. Gesells Ärzt Wien*. (1848) 5:64.

[50] Routh CH. On the causes of the endemic puerperal fever of Vienna. Med Chir Trans. (1849) 32:27–40. 10.1177/095952874903200103 20895917 PMC2104036

[51] Wieger F. Des moyens prophylactiques mis en U.S.A. ge au grand hôpital de Vienne contre l'apparition de la fièvre puerpérale. Gaz Méd Strasb. (1849) 9:99–105.

[52] Semmelweis IF. Die Aetiologie der Begriff und die Prophylaxis des Kindbettfiebers. Wien: C.A. Hartleben's Verlags-Expedition (1861).

[53] Semmelweis IF. Aetologie des Puerperalfiebers [A gyermekágyi láz kórtana] (1858). In: Gyori T, editor. Collected Works of Semmelweis. Budapest: Magyar Orvosi Könyvkiadó Társulat (1906). p. 29–56.

[54] Labarraque AG. Maniére de se servir du shlorure d'oxyde de sodium. et de désinfections, etc. Paris: Huzard (1825).

[55] Bencko V Schejbalová M. From Ignaz Semmelweis to the present: crucial problems of hospital hygiene. Ind Built Env. (2006) 15:3–7. 10.1177/1420326X06062362

[56] Meigs CD. On the Nature, Sign, and Treatment of Childbed Fever, etc. Philadelphia, PA: Blanchard and Lea. (1854).

[57] Lane HJ Blum N Fee E. Oliver Wendell Holmes (1809–1894) and Ignaz Phillipp Semmelweis (1818–1865): preventing the transmission of puerperal fever. Ame J Publ Health. (2010) 100:1008–9. 10.2105/AJPH.2009.185363 20395569 PMC2866610

[58] Nightingale F. Notes on Nursing: What It Is and What It Is Not. London: Harrison and Sons (1860).

[59] Attewell A. Florance Nightingale (1820–1910). Prospects. (1998) 28:151–66. 10.1007/BF02737786

[60] Pasteur L. Études sur le Vin: ses maladies causes qui les provoquent proceeds nouveaux pour le conservier et pour le viellir. Paris: A L'Imprimerie Impériale (1866).

[61] Smith KA. Louis Pasteur, the father of immunology? Front Immunol. (2012) 3:68. 10.3389/fimmu.2012.00068 22566949 PMC3342039

[62] Hurwitz B Dupree M. Why celebrate Joseph Lister? Lancet. (2012) 379:E39–40. 10.1016/S0140-6736(12)60245-1 22385682

[63] Lister J. Illustrations of the antiseptic system of treatment in surgery. Lancet. (1867) 90:668–9. 10.1016/S0140-6736(02)58116-2 11199534

[64] Koch R. Untersuchungen über Bakterien: V. Die Ätiologie der Milzbrand-Krankheit, begründet auf die Entwicklungsgeschichte des Bacillus anthracis. Coh Beit Biol Pflan. (1876) 2:277–310.

[65] Morton LT. Garrison and Morton's Medical Bibliography, 2nd ed. London: Grafton (1954).

[66] Lea AWW. Puerperal Infection. Oxford: Oxford University Press (1910).

[67] Smellie WA. A Treatise on the Theory and Practice of Midwifery, 2nd Edn. London: D. Wilson and T. Durham at Plato's Head in the Strand (1752).

[68] Cooper T. Proposals for teaching the art of midwifery: with a syllabus of the lectures. London: Middlesex Hospital (1767).

[69] Leake J. Practical Observations in the Childbed Fever. London: J. Walter (1772).

[70] White C. Treatise on the Management of Pregnant and Lying-in Women. London: Charles Dilly (1773).

[71] Churchill F. Essays on the Puerperal Fever and Other Diseases Peculiar to Women. London: Sydenham Society (1849).

[72] Gordon AA. A Treatise on the Epidemic Puerperal Fever of Aberdeen. London: G.G. And J Robinson (1795). 4595937

[73] Gould IM. Alexander Gordon, puerperal sepsis, and modern theories of infection control – Semmelweis in perspective. Lancet Inf. Dis. (2010) 10:275–8. 10.1016/S1473-3099(09)70304-4 20334850

[74] Denman T. An Introduction to the Practice of Midwifery. London: J. Johnson (1801).

[75] Zsoldos J. The French in Pápa, or the description of the Civil Hospitals in Pápa during 1809–1810. Tud Gyujt. (1817) 11:47–76. [In Hungarian].

[76] Varga M.M. János Zsoldos (1767–1832) first medical officer of the country of Veszprém. Comm Hist Artis Med. (1976) 80:27–47. [In Hungarian].

[77] Zsoldos J. Diaetetics or rules of procedures ot maintain health and prevent diseases. Gyor: Özvegy Streibig Józsefné (1814). p. 197–198. [In Hungarian].

[78] Zsoldos J. Diaetetics or rules of procedures ot maintain health and prevent diseases, 2nd ed. Pest: Trattner Tamás János (1818) pp. 150–159. [In Hungarian].

[79] Csillag I. A history of the Semmelweis concept in Hungarian medical literature. Orv Hetil. (1968) 109:874–7. [In Hungarian]. 4881928

[80] Zsoldos J. Egészség regulái. Mostan pedig a' Helvétziai vallástételt tartó Négy Fo Tiszteletu Superintendentziák' Egyházi fobb Konstitóriumának rendeletébol, Versekbe foglalta, és mint a' Négy Fo Tiszteletu Superintendentziákban, az apróbb Tanuló Gyermekek' számára megrendelt Oskolai Kézi Könyvet saját költéségn kinyomtatta Zsoldos Jakab. Gyor, Streibig Leopold: Gyor, Hungary (1817).

[81] Zsoldos J. Az egészség fentartásról való rendszabások (Diaetetica). Versekbe foglalta tiszt. Superintendentiák rendelésébol Fodor Gerson. Nádaskai András: Sárospatak, Hungary (1818).

[82] “The book of the sick women's notary from 1 April 1816 in Pápa (A beteg asszonyi nem ispitállyának jegyzokönyve 1816-ik esztendejei április 1-sejétol kezdve Pápánn). Hungary: Library of the Transdanubian Reformed Church District, Manuscript Repository (1860). p. 460.

[83] Jakab R. “The hospital of the sick woman” women's hospital in Pápa in the early 19th century. In: Berényi I, Géra E, Richly G, eds. “*Teach Us to Count Our Days.” Studies in Honor of the 70 Years Old László Kósa*. Budapest: ELTE Eötvös Kiadó (2014). p. 227–58.

[84] Othersen MJ Othersen HB. A history of handwashing: seven hundred years at a snail's pace. Pharos. (1987) 50:23–7. 3299403

[85] United Nations Children's Fund and World Health Organization. State of the World's Hand Hygiene: A Global Call to Action to Make Hand Hygiene a Priority in Policy and Practice. New York, NY: UNICEF (2021).

[86] Chattopadhyay A Sethi V Nagargoje VP Saraswat A Surani N Agarwal N. WASH practices and its association with nutritional status of adolescent girls in poverty pockets of eastern India. BMC Wom Health. (2019) 19:89. 10.1186/s12905-019-0787-1 31277634 PMC6612154

[87] Loveday H Wilson J. COVID-19: fear, explanation, action, unity and ingenuity and World Hand Hygiene Day. J Inf Prev. (2020) 21:80–2. 10.1177/1757177420921963 32494291 PMC7238693

[88] Holmes OW. Puerperal Fever as a Private Pestilence. Boston: Ticknor and Fields (1855). p. 59.

[89] Poczai P Santiago-Blay JA Sekerák J Szabó AT. How political repression stifled the nascent foundations of heredity research before Mendel in Central European sheep breeding societies. Philosophies. (2021) 6:41. 10.3390/philosophies6020041

[90] Poczai P Santiago-Blay JA Sekerák J Bariska I Szabó TA. Mimush sheep and the spectre of inbreeding: historical background for Festetics's organic and genetic laws four decades before Mendel's experiments in peas. J Hist Biol. (2022). 10.1007/s10739-022-09678-5 35670984 PMC9668798

[91] Poczai P. Heredity before Mendel. Boca Raton: CRC Press (2022). p. 86–96.

[92] Semmelweis IF. The difference of opinion between myself and the English doctors over puerperal fever (1860). In: Gyori T, editor. Collected works of Semmelweis. Budapest: Magyar Orvosi Könyvkiadó Társulat (1906). p. 59–73.

[93] Csillag I. New data on the history of the Semmelweis concept in Hungary. Comm Hist Artis Med. (1970) 55-56:201–6 [In Hungarian].28818505

[94] Wilkinson G. Epidemics: wash your hands! The asylum delivery and violent death of Professor Ignaz Philipp Semmelweis; and, the cursed Semmelweis reflex – psychiatry in history. Brit J Psych. (2021) 218:242. 10.1192/bjp.2020.238

[95] Gupta VK Saini C Oberoi M Kalra G Nasir MI. Semmelweis reflex: an age-old prejudice. World Neuros. (2020) 136:e119–25. 10.1016/j.wneu.2019.12.012 31837492

[96] Mortell M Balkhy HH Tannous EB Jong MT. Physician ‘defiance' towards hand hygiene compliance: is there a theory-practice-ethics gap? J Saud Heart Assoc. (2013) 25:203–8. 10.1016/j.jsha.2013.04.003 24174860 PMC3809478

[97] Makhni S Umscheid CA Soo J Chu V Bartlett A Landon E Marrs R. Hand hygiene compliance rate during the COVID-19 pandemic. JAMA Internal Med. (2021) 181:1006–8. 10.1001/jamainternmed.2021.1429 33900357 PMC8077041

[98] Ramlogan R Consoli D. Knowledge, understanding and the dynamics of medical innovation. In: Manchester Business School Working Paper, No. 539. Manchester: The University of Manchester, Manchester Business School (2008). Available online at: http://hdl.handle.net/10419/50694 (accessed September 29, 2022).

[99] Karvalics ZL. On the information history of medicine. Kaleidoscope. (2014) 5:25–37. [In Hungarian]. 10.17107/KH.2014.9.25-37

[100] Hall H. Handwashing in medicine: infrequent use of an ancient practice. Int J Psychosom. (1995) 42:44–7. 8582811

